# Src-mediated phosphorylation of the ribosome biogenesis factor hYVH1 affects its localization, promoting partitioning to the 60S ribosomal subunit

**DOI:** 10.1016/j.jbc.2022.102679

**Published:** 2022-11-10

**Authors:** Ashley A. DaDalt, Christopher A. Bonham, Griffin P. Lotze, Adrian A. Luiso, Panayiotis O. Vacratsis

**Affiliations:** Department of Chemistry and Biochemistry, University of Windsor, Windsor, Ontario, Canada

**Keywords:** dual-specificity phosphoprotein phosphatase, DUSP12, hYVH1, mass spectrometry, phosphotyrosine, proteomics, Src, YVH1, AB, ammonium bicarbonate, ACN, acetonitrile, BSA, bovine serum albumin, CHX, cycloheximide, DEPC, diethyl pyrocarbonate, DIA, data-independent acquisition, DUSP, dual-specificity phosphatase, EF2, elongation factor 2, EIF6, eukaryotic translation initiation factor 6, FA, formic acid, hYVH1, human YVH1, IFRD, interferon-related developmental regulator, MS, mass spectrometry, PCC, Pearson correlation coefficient, RACK1, receptor of activated protein C kinase 1, RNP, ribonucleoprotein, TIA-1, T-cell intracellular antigen-1, YB-1, Y-box binding protein 1, YVH1, yeast VH1-related phosphatase, ZBD, zinc-binding domain

## Abstract

Yeast VH1-related phosphatase (YVH1) (also known as DUSP12) is a member of the atypical dual-specificity phosphatase subfamily. Although no direct substrate has been firmly established, human YVH1 (hYVH1) has been shown to protect cells from cellular stressors, regulate the cell cycle, disassemble stress granules, and act as a 60S ribosome biogenesis factor. Despite knowledge of hYVH1 function, further research is needed to uncover mechanisms of its regulation. In this study, we investigate cellular effects of a Src-mediated phosphorylation site at Tyr^179^ on hYVH1. We observed that this phosphorylation event attenuates localization of hYVH1 to stress granules, enhances shuttling of hYVH1 to the nucleus, and promotes hYVH1 partitioning to the 60S ribosomal subunit. Quantitative proteomics reveal that Src coexpression with hYVH1 reduces formation of ribosomal species that represent stalled intermediates through the alteration of associating factors that mediate translational repression. Collectively, these results implicate hYVH1 as a novel Src substrate and provide the first demonstrated role of tyrosine phosphorylation regulating the activity of a YVH1 ortholog. Moreover, the ribosome proteome alterations point to a collaborative function of hYVH1 and Src in maintaining translational fitness.

In 1992, Guan *et al.* (1) cloned and characterized the yeast VH1-related phosphatase (YVH1), marking it as one of the earliest eukaryotic dual-specificity phosphatases (DUSPs) identified. The mRNA levels of YVH1 were found to be dramatically induced by nitrogen starvation and low temperatures. In addition, knockdown of the *yvh1* gene in yeast exhibited a slow growth phenotype, with defects in glycogen accumulation and spore maturation (2). Following these studies in yeast, the human ortholog (hYVH1, also known as DUSP12) was identified and shown to share 31% sequence identity with YVH1 (3). Interestingly, the *yvh1* gene has now been shown to be widely conserved throughout evolution in eukaryotes from yeast to humans (1, 2, 3).

YVH1 orthologs are characterized by possessing an N-terminal tyrosine phosphatase domain, which possesses the characteristic CX_5_R motif, and a C-terminal zinc-binding domain (ZBD). These two domains are connected by a linker region of unknown function. Initial studies on hYVH1 have shown its relevance in various cellular functions, including cell survival and the cell cycle (4, 5). When investigating further into the capability of hYVH1 to protect cells from oxidative stress induced by H_2_O_2_, it was revealed that the ZBD acts as a redox sensor, capable of forming intramolecular disulfide bonds (6). This allows for hYVH1 to avoid irreversible inactivation during severe oxidative stress by protecting the catalytic cysteine residue, again highlighting the importance of this unique ZBD. Once the stress is removed and reducing conditions have been established, zinc ejection is readily reversible (and required) to recover the intrinsic phosphatase activity of hYVH1 *in vitro* (6).

A central aspect of YVH1 biology has been the revelation of its role in 60S ribosome biogenesis. In yeast, YVH1 has been shown to act as a critical transacting factor responsible for exchanging the placeholder Mrt4 for the P_0_ stalk protein. Association of P_0_ is required to complete maturation of the 60S ribosomal subunit leading to efficient 80S ribosome formation. In addition to its role in 60S ribosome biogenesis, an interactome analysis recently discovered that hYVH1 associates with multiple ribonucleoprotein (RNP) complex proteins, including stress granules (7). Upon further investigation, it was demonstrated that hYVH1 functions as a stress granule disassembly factor (7).

Although, increasing evidence points to a role for hYVH1 in RNP remodeling, less is known how hYVH1 is regulated temporally and spatially. In this study, we demonstrate that the tyrosine kinase Src is able to phosphorylate hYVH1 within its linker region, leading to increased nuclear translocation and 60S ribosome association. Quantitative proteomics discovered that coexpression of hYVH1 and Src leads to protein alterations on the 80S ribosome consistent with a role in translational fitness demonstrating for the first time that hYVH1 is regulated by tyrosine phosphorylation.

## Results and discussion

### Phosphorylation site identification on hYVH1

**Figure 1:**
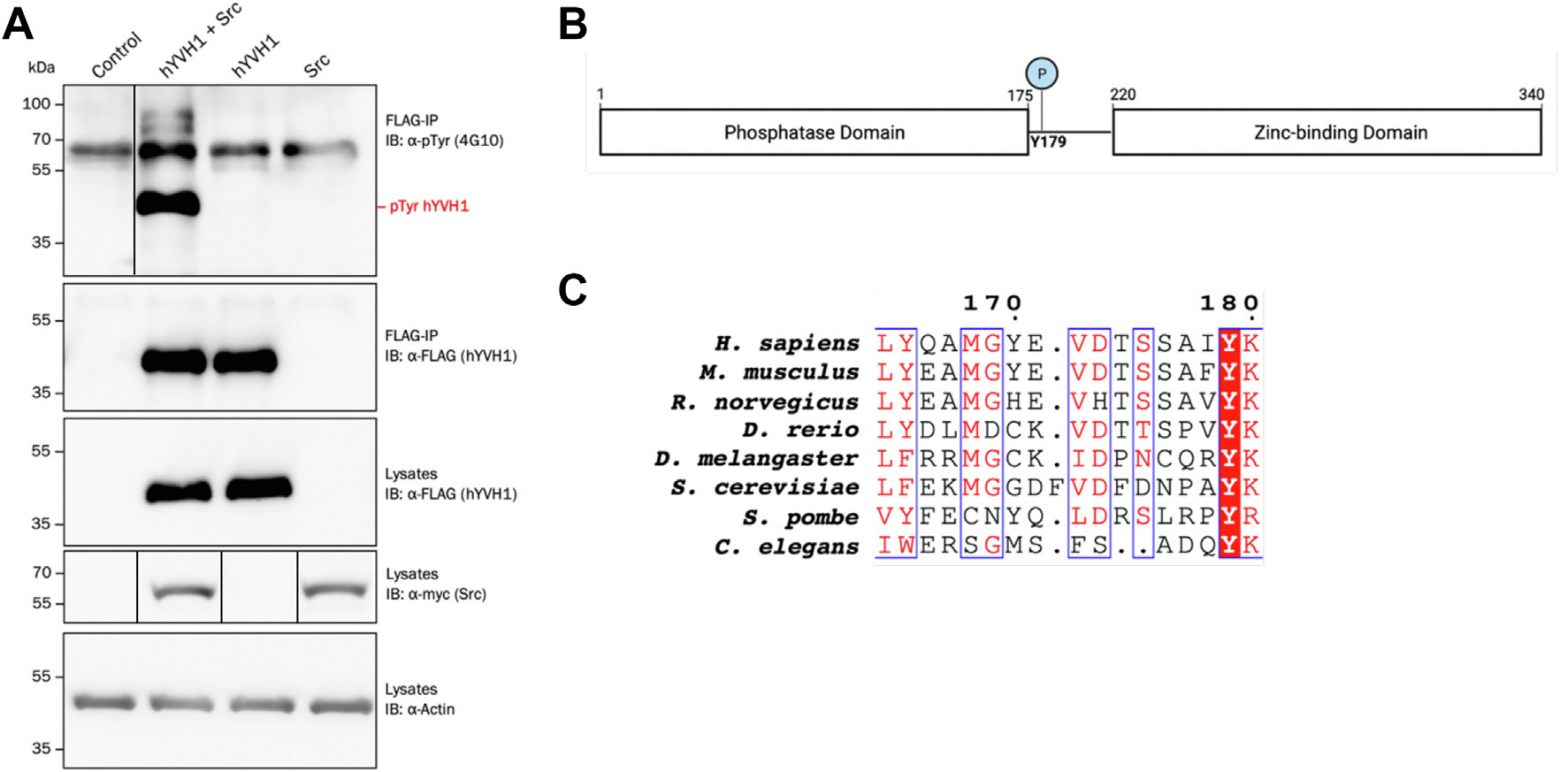
**Src phosphorylates hYVH1 on residue Tyr 179.***A*, HeLa cells expressing FLAG-hYVH1 and myc-Src Y530F were lysed and subjected to FLAG immunoprecipitation and immunoblot analysis. Antiphosphotyrosine (4G10) blot depicting an increase in hYVH1 tyrosine phosphorylation when coexpressed with Src is shown (*top panel*). *Boxed lines* on blots indicate where irrelevant sample lanes were cropped out to simplify blots and line up relevant samples. *B*, schematic representation of the hYVH1 sequence showing the location of the mapped phosphorylation site Tyr^179^ with respect to its domain structure. *C*, multiple sequence alignment of the tryptic peptide corresponding to amino acid residues 163 to 180 of hYVH1. Regions of conservation across species are highlighted including the phosphorylation site Tyr^179^. hYVH1, human YVH1.

Previously, our laboratory investigated hYVH1 complexes using affinity chromatography–based interactome techniques (7). During these experiments, we reproducibly observed low-confidence peptides assigned to the tyrosine kinase Src. Although these data did not pass our confidence criteria under the conditions used, we were interested in directly investigating if Src was able to phosphorylate tyrosine residues on hYVH1. To examine this, HeLa cells expressing FLAG-hYVH1 and myc-Src Y530F were subjected to FLAG-immunoprecipitation and analyzed by antiphosphotyrosine immunoblotting (Fig. 1*A*). The results demonstrated that when coexpressed with Src, robust tyrosine phosphorylation was observed. We also detected low level but reproducible tyrosine phosphorylation on endogenous hYVH1 when HeLa cells were stimulated with epidermal growth factor (Fig. S1). Interestingly, tyrosine phosphorylation of endogenous hYVH1 was decreased when cells were treated with a specific inhibitor to inactivate endogenous Src in HeLa cells (Fig. S1). To confirm the phosphotyrosine immunoblot analysis and map the phosphotyrosine residue(s) being modified, mass spectrometry (MS) was performed on hYVH1 immunoprecipitants as detailed in the “Experimental procedures” section. Initial measurements employing data-independent acquisition (DIA) MS parameters identified a monophosphorylated peptide at *m/z* 1017.9^+2^ corresponding to amino acid residues 164 to 180 of hYVH1. The corresponding unphosphorylated peptide was also identified at *m/z* 977.9^+2^. As this peptide contains six phosphorylatable residues, including three Tyr residues, the sample was reanalyzed using high-definition selected reaction monitoring, a targeted MS technique that can increase sensitivity and peptide fragmentation (8). Upon analysis of the fragment ion series, phosphorylation of residue Tyr^179^ was confidently assigned because of the most intense fragment ion peak corresponding to a y_2_ C-terminal ion consisting of a phosphoTyr-Lys dipeptide (Fig. S2). The masses of the other fragment ions supported the assignment of the phosphorylation site to Tyr^179^. We also observed tyrosine phosphorylation of hYVH1 following an *in vitro* kinase assay where myc-Src was immunoprecipitated from HeLa cells, and purified recombinant hYVH1 C115S (catalytically inactive mutant) was added as the substrate (Fig. S3). Importantly, when analyzed by high-definition selected reaction monitoring MS, this *in vitro* phosphorylated hYVH1 was found to occur on Tyr^179^ (Fig. S3). Moreover, a kinase assay using purified recombinant WT Src and purified recombinant hYVH1 C115S revealed that Src can directly phosphorylate hYVH1 *in vitro*. When examining the primary sequence and three-dimensional structure of hYVH1, Tyr^179^ resides in a helix located at the N-terminal portion of the linker region between the phosphatase and ZBDs (Fig. 1*B*) (9). Interestingly, this tyrosine residue is conserved in all YVH1 orthologs examined, including yeast, suggesting it may play an important functional role (Fig. 1*C*).

### Phosphorylation of hYVH1 reduces stress granule disassembly

**Figure 2:**
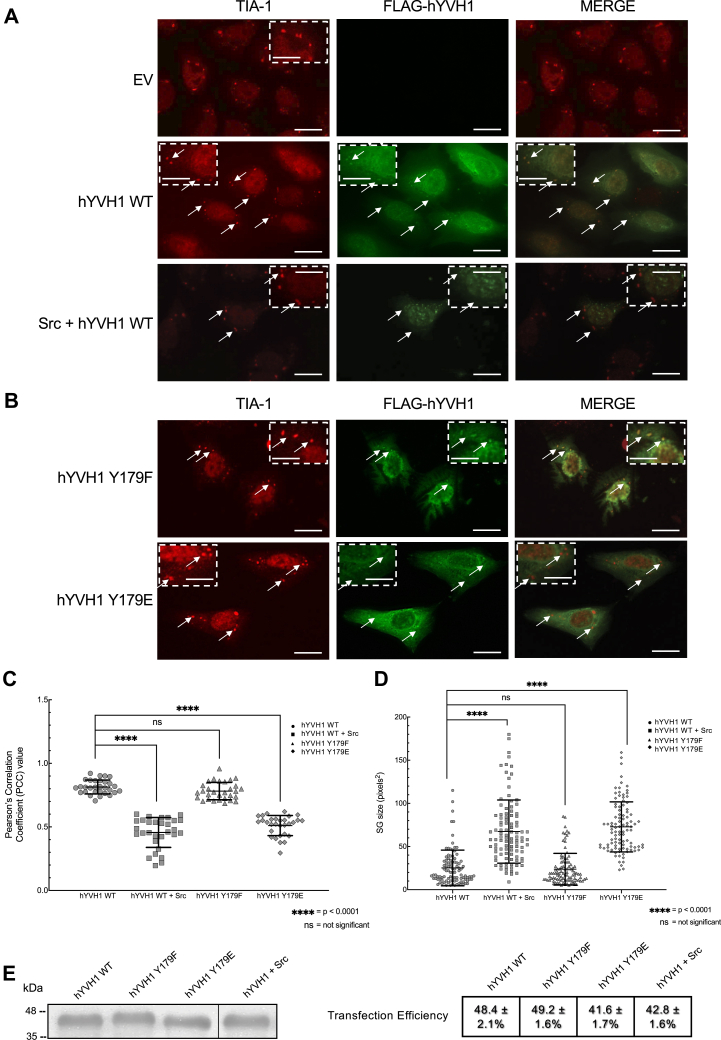
**Reduced hYVH1 colocalization with stress granules in response to Src expression.** Immunofluorescent microscopy images of HeLa cells with TIA-1 used to mark stress granules (*red*) induced by arsenic stress. Colocalization was examined using a FLAG antibody to detect FLAG-hYVH1 (*green*). *White arrows* are used to indicate granules of interest. The scale bars represent 25 μm. *Boxed regions* are magnified with scale bars that represent 12.5 μm. *A*, *top panel* displays empty vector (EV) control. *Second panel* displays representative cells expressing FLAG-hYVH1 (WT), with hYVH1-positive granules colocalizing with stress granules. *Third panel* contains FLAG-hYVH1 coexpressed with Src. *B*, colocalization of phosphomimetic hYVH1 variants with stress granules. *Top panel* displays cells expressing hYVH1 Y179F, whereas the *bottom panel* shows hYVH1 Y179E. *C*, Pearson correlation coefficients to quantify the extent of colocalization between hYVH1 and stress granules using ImageJ. Means ± SD of three independent experiments (n = 30 cells) are shown. A Student’s *t* test was used to calculate *p* values and compared with FLAG-hYVH1, with differences considered statistically significant at *p* < 0.0001 (∗∗∗∗). *D*, stress granule size in pixels^2^/granule. Means ± SD of three independent experiments (n = 100 stress granules) are shown. A Student’s *t* test was used to calculate *p* values and compared with FLAG-hYVH1, with differences considered statistically significant at *p* < 0.0001 (∗∗∗∗). *E*, representative anti-FLAG immunoblot showing equal expression levels of FLAG-hYVH1 variants in HeLa cells under arsenic stress. *Boxed line* on blot indicates where irrelevant sample lanes were cropped out to simplify blot. *F*, transfection efficiency of FLAG-hYVH1 variants determined by manually counting FLAG-hYVH1–positive cells divided by total number of Hoechst-stained cells. Values represent the means ± SD of three independent experiments (n = 150 cells). hYVH1, human YVH1; TIA-1, T-cell intracellular antigen-1.

We have previously shown that hYVH1 functions as a stress granule disassembly factor (7). Using a combination of hYVH1 deletion variants and time-course knockdown/rescue experiments, we demonstrated that hYVH1 readily colocalizes to T-cell intracellular antigen-1 (TIA-1) positive stress granules in response to arsenic stress. While knockdown of hYVH1 did not disrupt formation of stress granules, disassembly was impaired as observed by a notable increase in stress granule size during stress recovery. Furthermore, stress granule size was significantly reduced when hYVH1 was overexpressed in HeLa cells following arsenic stress. Therefore, it was of interest to determine if Src-mediated tyrosine phosphorylation of hYVH1 would affect the ability of hYVH1 to localize to TIA-1-positive stress granules. Stress granules were induced by arsenic stimulation of HeLa cells, and immunofluorescence microscopy was used to observe TIA-1-positive stress granules (10, 11). As expected, hYVH1 expressed alone localizes to stress granules in response to arsenic treatment (Fig. 2, *A* and *C*). However, upon Src coexpression, hYVH1 displayed reduced targeting to stress granules (Fig. 2, *A* and *C*), suggesting that phosphorylation of Tyr^179^ may direct hYVH1 away from stress granules. Stress granule disassembly can be inferred by stress granule size, as stress granules get smaller in response to stress granule disassembly. Interestingly, larger stress granules are observed when hYVH1 is coexpressed with Src compared with when expressed alone (Fig. 2, *A* and *D*), further supporting that tyrosine phosphorylation of hYVH1 attenuates localization to stress granule particles and the role of hYVH1 in stress granule disassembly.

Phosphomimetics of Tyr^179^ to represent constitutively unphosphorylated (Y179F) and phosphorylated (Y179E) hYVH1 were synthesized using site-directed mutagenesis to complement the Src coexpression data. Consistently, the hYVH1 Y179F variant displayed a similar stress granule colocalization pattern and produced similar stress granule sizes as WT hYVH1, suggesting that the dephosphorylated form of hYVH1 facilitates the stress granule disassembly function of hYVH1 (Fig. 2, *B*–*D*). Interestingly, hYVH1 Y179E was significantly impaired at localizing to stress granules, analogous to Src coexpressed with hYVH1. Moreover, stress granules in cells expressing hYVH1 Y179E were larger in size (Fig. 2*B*).

To quantify the aforementioned qualitative observations, image analysis was performed *via* ImageJ (NIH) using the JACoP plugin. First, the localization of hYVH1 to stress granules was analyzed on n = 30 cells from three independent experiments, and Pearson correlation coefficients (PCCs) were calculated and plotted in Figure 2*C*. A PCC of 1.0 indicates complete positive correlation, 0 meaning no correlation, and a value of −1.0 indicating negative correlation. The discovered qualitative observations were confirmed statistically as stress granule colocalization with WT hYVH1 and the hYVH1 Y179F mutant yielding relatively high PCCs of 0.814 and 0.782, respectively. When hYVH1 was coexpressed with Src and for mutant hYVH1 Y179E, much lower PCC values were obtained at 0.456 and 0.510, respectively, indicating loss of colocalization with stress granules (Fig. 2*C*). In addition, after measuring stress granule size for each sample (n = 100 stress granules), the stress granules were statistically smaller when hYVH1 WT or hYVH1 Y179F were expressed (means of 25.16 and 23.73 pixels^2^, respectively) and larger for hYVH1 + Src and hYVH1 Y179E (means of 67.32 and 72.76 pixels^2^, respectively) (Fig. 2*D*). Taken together, these results suggest that Src-mediated phosphorylation of hYVH1 impairs its ability to associate with stress granules and participate in their disassembly.

### Src phosphorylation alters subcellular localization of hYVH1

**Figure 3:**
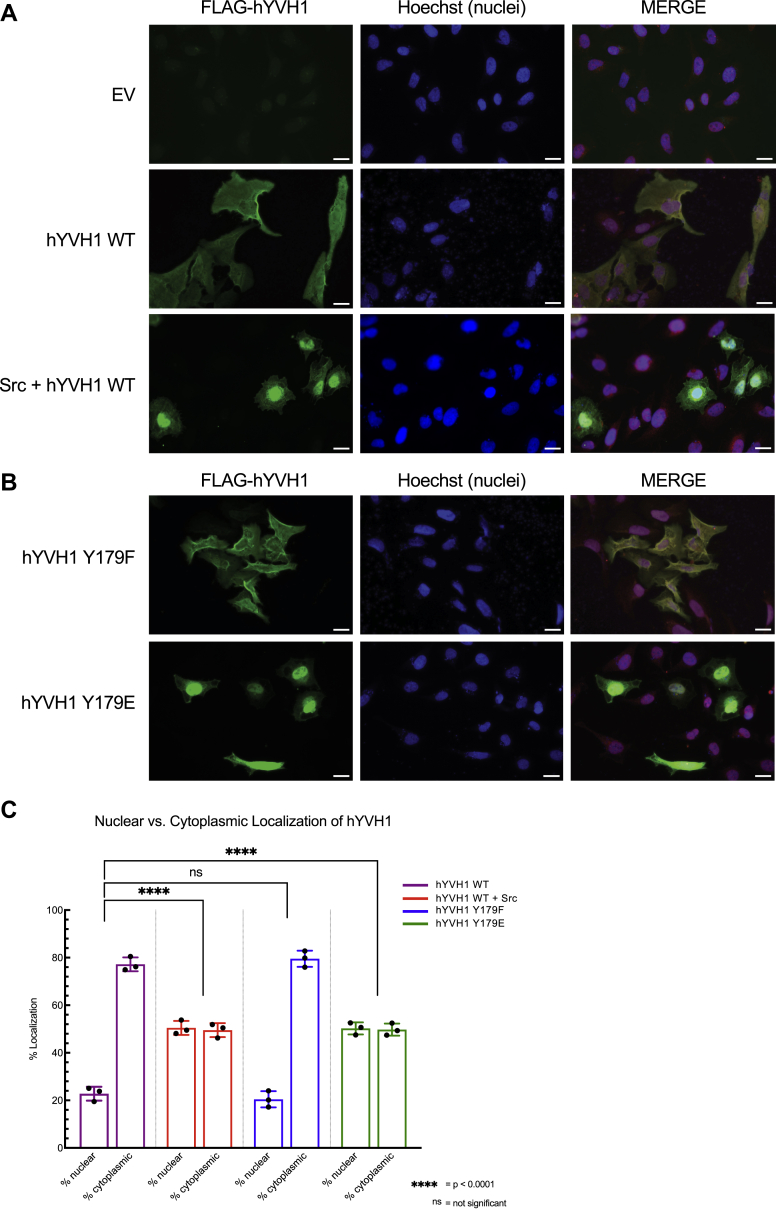
**hYVH1 subcellular localization in response to Src expression.** HeLa cells expressing the indicated hYVH1 variants were analyzed by immunofluorescent microscopy. Cells were probed with anti-FLAG to detect FLAG-hYVH1 (*green*), nuclei were stained with Hoechst (*blue*), and a merged depiction of these images is shown. The scale bars represent 25 μm. *A*, *top panel* displays empty vector (EV) control cells. *Second panel* displays representative cells with overexpressed FLAG-hYVH1, with prominent cytoplasmic localization. *Third panel* displays representative cells coexpressing FLAG-hYVH1 and Src, which displays higher nuclear localization. *B*, *top panel* displays cells expressing FLAG-hYVH1 Y179F, showing largely cytoplasmic localization, whereas the *bottom panel* shows FLAG-hYVH1 Y179E and its predominant nuclear localization. *C*, *boxed* plot illustrating the frequency of subcellular localization in % nuclear and % cytoplasmic for the various hYVH1 constructs, calculated *via* ImageJ software. Means ± SD of three independent experiments (n = 30 cells) are shown. A Student’s *t* test to calculate *p* values and compared with hYVH1 WT, with differences considered statistically significant at *p* < 0.0001 (∗∗∗∗).hYVH1, human YVH1.

The observation that Src-mediated phosphorylation attenuates the localization of hYVH1 to stress granules suggests that a consequence of Tyr^179^ phosphorylation is to alter the subcellular localization of hYVH1. It is well documented that YVH1 orthologs are capable of shuttling between the nucleus and cytoplasm (12, 13). It is thought that this nuclear/cytoplasmic shuttling phenotype is indicative of the role of YVH1 in 60S ribosome biogenesis, where it is postulated that YVH1 associates with the maturing 60S subunit in the nucleus and disassociates from the 60S subunit in the cytoplasm (12, 13). As protein phosphorylation is a common mechanism regulating subcellular localization of a protein (14), and to complement the finding that Tyr^179^ phosphorylation reduces hYVH1 stress granule targeting, we were interested in determining if Src-mediated phosphorylation of hYVH1 affected the steady-state localization of hYVH1. WT hYVH1 expressed alone in HeLa cells displayed a nuclear/cytoplasmic localization pattern with greater concentration in the cytoplasm and perinuclear region (Fig. 3*A*). Conversely, upon coexpression with Src, hYVH1 localization pattern dramatically shifts to a higher nuclear expression pattern (Fig. 3*A*). Examination of the phosphomimetic hYVH1 mutants supported the Src coexpression data. The localization pattern of the hYVH1 Y179F variant resembled WT hYVH1, whereas the Y179E variant displayed a more nuclear localization pattern similar to hYVH1 coexpressed with Src (Fig. 3*B*). Using the Intensity Ratio Nuclei Cytoplasm plugin on ImageJ, the translocation data were quantified (Fig. 3*C*). WT hYVH1 displayed a 21.64% nuclear and 78.36% cytoplasmic pattern, whereas hYVH1 Y179F had a similar 21.03% nuclear and 78.97% cytoplasmic localization pattern. In contrast, hYVH1 coexpressed with Src displayed a 52.15% nuclear and 47.85% cytoplasmic localization pattern, and likewise, hYVH1 Y179E displayed a 49.64% nuclear and 50.36% cytoplasmic localization pattern. Collectively, these results indicate that Src-mediated phosphorylation of hYVH1 may regulate the shuttling hYVH1 into the nucleus. The observation that Src-mediated phosphorylation affects the nuclear localization of hYVH1 is compelling when we consider its role in ribosome biogenesis. The recycling of transacting factors such as hYVH1 back into the nucleus has been shown to be a critical feature for maintaining a steady-state rate of ribosome biogenesis (12, 13). Therefore, our observation that Src-mediated phosphorylation results in higher nuclear localization may indicate that this phosphorylation event increases the rate of hYVH1 ribosome recycling.

### Src phosphorylation of hYVH1 increases association with 60S ribosomal subunit

There is evidence that nuclear/cytoplasmic shuttling is a key feature of the hYVH1-mediated maturation of the 60S ribosomal subunit (12, 13). Specifically, YVH1 in yeast has been shown to act as a transacting/recycling factor to displace Mrt4 on the pre-60S subunit in the nucleus and is subsequently replaced by the cytoplasmic homolog P_0_ once the pre-60S reaches the cytoplasm. At this point, YVH1 is liberated and free to be recycled back into the nucleus for another round of 60S subunit biogenesis. Coupled to the observation that the nuclear/cytoplasmic distribution of hYVH1 was altered in response to Src coexpression, it was important to determine if tyrosine phosphorylation impacts hYVH1 association with the 60S subunit. Furthermore, we were interested in optimizing ribosomal profiling conditions that would allow us to explore proteome alterations induced by coexpression of hYVH1 and Src.

**Figure 4:**
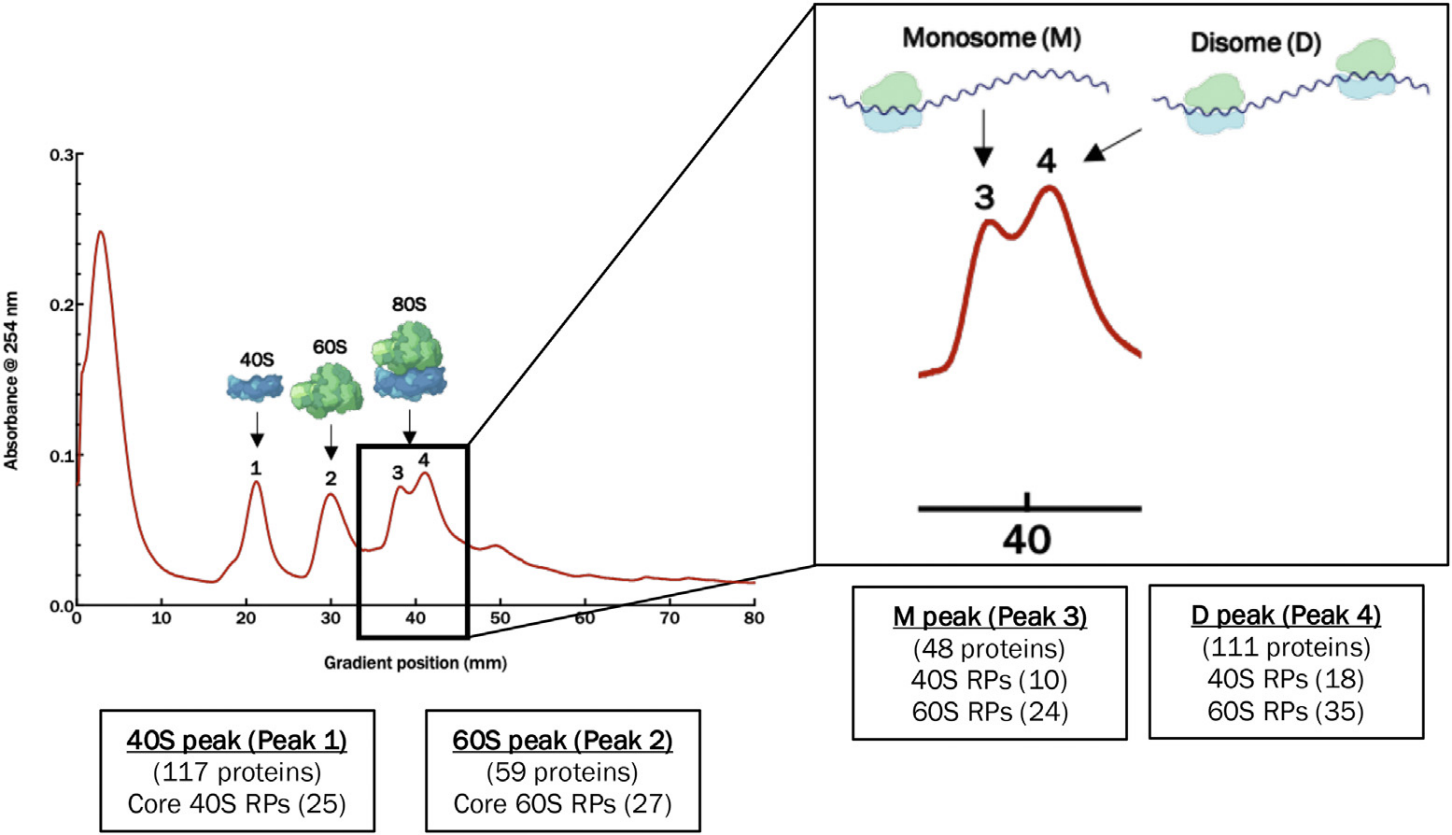
**Proteomic profiling of ribosome peaks.** HeLa whole cell lysate was applied to a 5 to 40% sucrose gradient and subjected to ultracentrifugation to separate ribosomal subunits. Samples were fractionated, and the absorbance at 254 nm was recorded. Fractions specific to the four peaks of interest from a representative control sample were subject to in-gel digestion and mass spectrometry analysis (Table S1). Peaks 1 and 2 were unambiguously confirmed as the 40S and 60S ribosomal subunits, respectively, as indicated by the core proteins identified. The 80S split peak (peaks 3 and 4) was confirmed as the mature 80S ribosome, containing 40S and 60S proteins with a notably higher abundance in peak 4, consistent with the presence of an additional mature ribosome (disome).

To initiate these objectives, large-scale ribosomal profiling experiments were optimized using a workflow that involved isolation of the ribosomal subunits by density gradient ultracentrifugation through a 5 to 40% sucrose gradient that was mechanically prepared and fractionated on a BioComp Gradient Master system. RNPs appear at the lowest sucrose percentage (the top of the gradient), followed by the 40S, 60S, 80S ribosomal subunits, and last the polysomes. The gradient fractions are collected while the absorbance rRNA at 254 nm is simultaneously measured. This creates a ribosomal profile that is then analyzed and subject to comparisons between samples of interest. Under our analytical setup and cell culture conditions, a typical ribosomal profile from HeLa cells was produced (Fig. 4). Interestingly, a partially resolved double peak on the gradient profile consistent with the monosome 80S fraction was reproducibly observed. We speculated that these fractions represented monosomes (one 80S subunit per mRNA molecule) and disomes (two 80S subunits per mRNA molecule). To confirm, the fractionated samples corresponding to each of the four peaks were analyzed by data-independent acquisition MS. Importantly, the measurements unambiguously confirmed that peaks 1 and 2 represent the 40S and 60S ribosomal subunits, respectively (Fig. 4 and Table S1). The proteomic profiling analysis for peaks 3 and 4, assumed to correspond to the 80S, confirmed that peak 3 contains an abundance of 40S and 60S core ribosomal proteins. This indicates that peak 3 corresponds to the 80S monosome. Peak 4 also contained every core ribosomal protein identified in peak 3 as well as additional core ribosomal proteins. Moreover, the relative abundance of all peptides/proteins was substantially higher in peak 4 fractions, suggesting that the additional core ribosomal proteins identified was due to higher concentration of ribosomal proteins in the sample. This is consistent with peak 4 representing the disome form of the mature ribosome and indicates that our chromatographic fractionation method was able to partially resolve monosome and disome ribosomal species. In addition to confidently characterizing the ribosomal subunit peaks, proteomic profiling of the fractions also allowed for assessing the purity of the ribosomal subunits from other subcellular entities. Thus, sucrose gradient density centrifugation provides sufficient resolving power to isolate the ribosomal segment of the proteome that can be used to explore hYVH1-mediated regulation of RNP dynamics.

**Figure 5:**
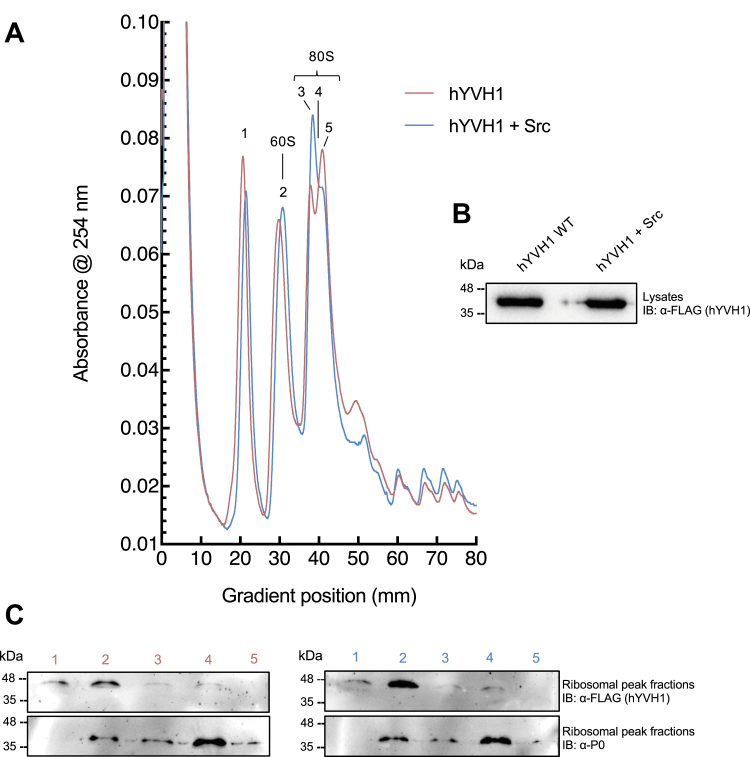
**Src coexpression increases hYVH1 association with 60S ribosome subunit.***A*, HeLa whole cell lysate from hYVH1 and hYVH1 + Src transfected cells were applied to a 5 to 40% sucrose gradient and subjected to ultracentrifugation to separate ribosomal subunits. The shown profiles are representative of five independent experiments (n = 5). *B*, representative FLAG-hYVH1 immunoblot showing input expression levels of FLAG-hYVH1 in whole cell lysates prior to sucrose gradient fractionation. *C*, immunoblot analysis of indicated fractions 1 to 5 were probed with anti-FLAG antibody to detect FLAG-hYVH1 (*top panel*) and anti-P_0_ antibody to detect the 60S core ribosomal protein P_0_ (*bottom panel*). hYVH1, human YVH1.

Upon optimization of the density fractionation method and identification of the profile peaks, we next probed the ribosomal fractions for hYVH1 when expressed alone or coexpressed with Src. Based on the results where Src attenuated association of hYVH1 with stress granules, one possibility is that Src-mediated phosphorylation may also diminish hYVH1 association with the 60S ribosomal subunit. Alternatively, the nuclear/cytoplasmic data where Src expression increased the amount of hYVH1 observed in the nucleus may signify a more efficient recycling of hYVH1 for its ribosome biogenesis function in response to Tyr^179^ phosphorylation. Interestingly, analysis of the fractionated samples by immunoblotting revealed that when hYVH1 is coexpressed with Src, the amount of hYVH1 in the 60S subunit sample was substantially higher (Fig. 5*C*). This observation was reproducible in multiple biological replicates (n = 3) suggesting that Src-mediated phosphorylation enhances targeting of hYVH1 to the 60S ribosomal subunit.

**Figure 6:**
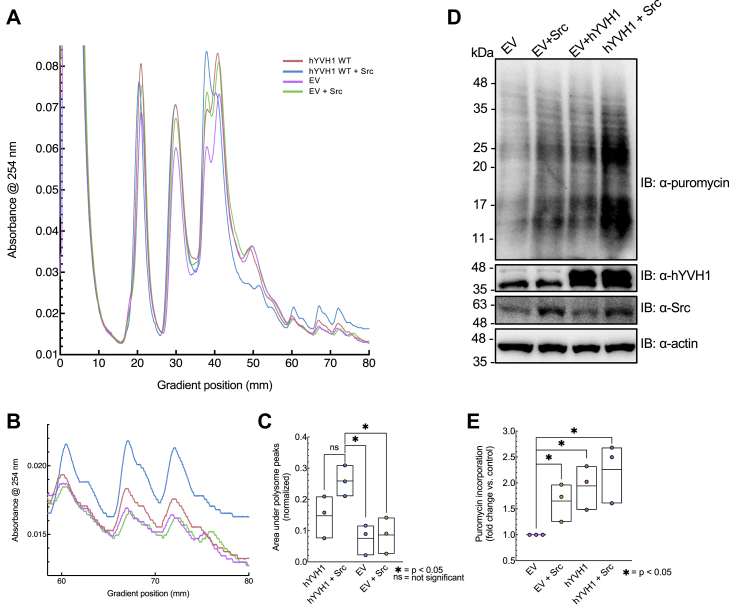
**Reduced disome fraction and increased translational rate in cells expressing hYVH1 and Src.** HeLa whole cell lysates were applied to a 5 to 40% sucrose gradient and subjected to ultracentrifugation to separate ribosomal subunits. The shown profiles are representative of three independent experiments (n = 3). *A*, overlay of all four samples: empty vector (EV) control (*purple*), EV + Src (*green*), FLAG-hYVH1 (*pink*), and FLAG-hYVH1 + Src (*blue*) ribosomal profiles. Highlighted by the *black box* is the difference in intensities between the monosome and disome peaks. *B*, overlay of all four profiles, zoomed in to examine the heavy polysomes. Evident is the higher peak intensities in the hYVH1 + Src sample (*blue*) and lower peak intensities of both EV control (*purple*) and EV + Src (*green*) samples. *C*, heavy polysome peaks were quantified by measuring the area under the curves using ImageJ software. Values were normalized to the areas calculated for the corresponding 40S peaks. Means ± SD of three independent experiments (n = 3) are shown. A Student’s *t* test to calculate *p* values and compared with hYVH1 + Src, with differences considered statistically significant at *p* < 0.05 (∗). *D*, representative images of puromycin incorporation as measured by antipuromycin immunoblotting. *E*, puromycin incorporation was quantified by densitometry measurements using ImageJ software. Data values were normalized to actin levels. Means + SD of three independent experiments (n = 3) are shown. A Student's *t* test was performed to calculate *p* values and compared with EV control, with differences considered statistically significant at *p* < 0.05 (∗). hYVH1, human YVH1.

Another compelling observation in the hYVH1 + Src sample was the notable decrease in the disome peak when compared with hYVH1 or Src expressed alone (Figs. 5*A* and 6*A*). This result is intriguing as a portion of disome structures represent stalled ribosomes resulting in translational pausing (15). Moreover, we also reproducibly observed an increase in heavy polysome peaks when Src was coexpressed with hYVH1 (Fig. 6, *B* and *C*). Quantitation of the area under the curves of the heavy polysome peaks for all the biological replicates (n = 3) normalized to 40S peak areas revealed a statistically significant increase in the levels of heavy polysomes in cells coexpressing hYVH1 and Src compared with control. To more directly examine effects on translational rates, we performed the SUnSET method (16), which measures the incorporation of puromycin into synthesizing polypeptides with use of a puromycin-specific antibody (Fig. 6, *D* and *E*). We observed a minor increase in puromycin incorporation in cells expressing Src alone (+1.6-fold) and hYVH1 alone (+1.9-fold). Interestingly, cells coexpressing both hYVH1 and Src displayed the greatest increase in puromycin incorporation (+2.6-fold) compared with control. Taken together, our results are consistent with the proposal that Src-mediated phosphorylation may recycle hYVH1 to the nucleus, facilitating its targeting to the 60S ribosomal subunit resulting in a state of higher translational fitness. To complement and validate these findings, we were interested in exploring potential proteomic differences induced by Src-mediated phosphorylation of hYVH1; specifically, at the monosome and disome.

### Quantitative proteomic analysis of monosome and disome structures

**Figure 7:**
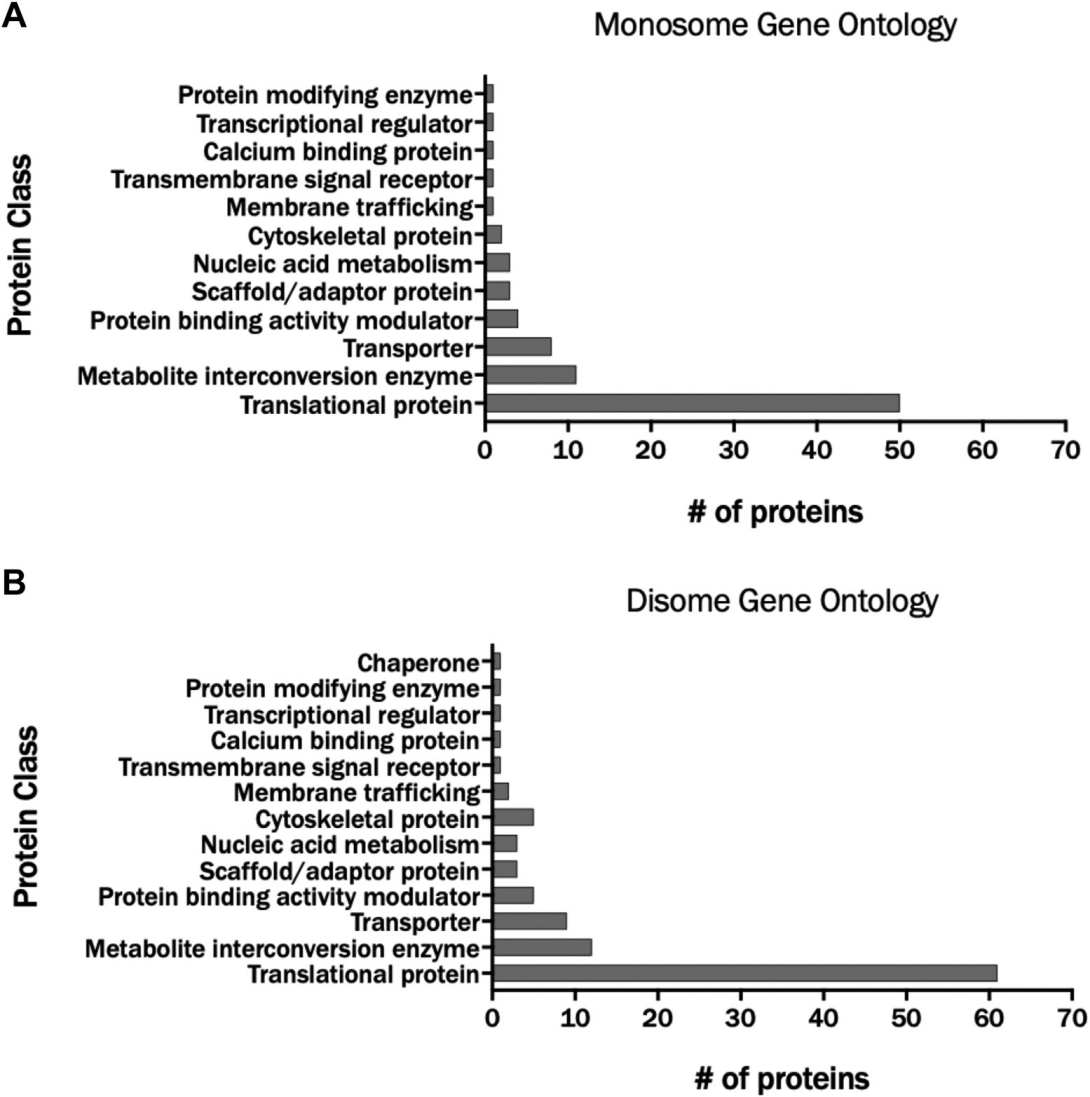
**Gene Ontology designations for monosome and disome proteins.** Proteomic analysis was performed on six biological replicates of ribosomal samples and separated into their respective protein classes. *A*, Gene Ontology breakdown for the monosome peak. *B*, Gene Ontology breakdown for the disome peak. Gene Ontology was obtained using the Panther classification system (http://www.pantherdb.org). In both samples, there is a significantly higher number of proteins related to translation compared with all other categories.

To obtain the proteomic alterations occurring during the distinct change in the monosome–disome chromatographic peak pattern upon coexpression of hYVH1 and Src, we compared this phenotype at the protein level with the empty vector control sample using label-free quantitative proteomics. Following sucrose gradient fractionation, samples corresponding to the monosome and disome were prepared for MS analysis using the short stack SDS-PAGE approach described in the “Experimental procedures” section. Generated tryptic peptides were separated by nano liquid chromatography using an 85 min acetonitrile (ACN) gradient and electrosprayed directly into the mass spectrometer. Mass analysis was performed in DIA mode, and ion mobility separation was used to increase resolution and improve protein identification yields (17). Samples were generated from six biological replicates (three from empty vector transfected control and three from hYVH1 + Src cotransfected HeLa cells). Altogether, 107 proteins in the monosome samples (Table S2) and 154 proteins in the disome samples (Table S3) were confidently identified. Gene Ontology (www.pantherdb.org) of the proteomic data confirmed that the vast majority of proteins identified were ribosomal proteins and regulators of translation (Fig. 7).

For quantitation purposes, only proteins that were identified with ≥3 unique peptides were considered. A Student's *t* test was used to determine protein alterations that were statistically significant (*p* < 0.05). Application of the Student's *t* test identified 23 and 32 putative significant protein alterations in the monosome samples and disome samples, respectively. Additional criteria were employed to provide enhanced statistical rigor. We employed ANOVA of all technical replicates with a *p* value cutoff of <0.001 and considered alterations significant if the fold change difference was at least >1.5. Collectively, statistical analysis of the proteomic data yielded 10 proteins in the monosome sample and 9 proteins in the disome sample that displayed altered protein levels in response to hYVH1 and Src coexpression. For the disome sample, all nine of the statistically significant protein alterations were the result of protein levels being reduced in response to hYVH1–Src coexpression. This result is consistent with the observed decrease in the 254 nm chromatographic peak when hYVH1 and Src are coexpressed. For the monosome sample, the levels of six proteins were reduced and four proteins elevated in response to hYVH1–Src coexpression. Importantly, the protein levels of the core ribosomal proteins were statistically similar between both conditions, providing additional confidence that the observed protein alterations are valid.

**Figure 8:**
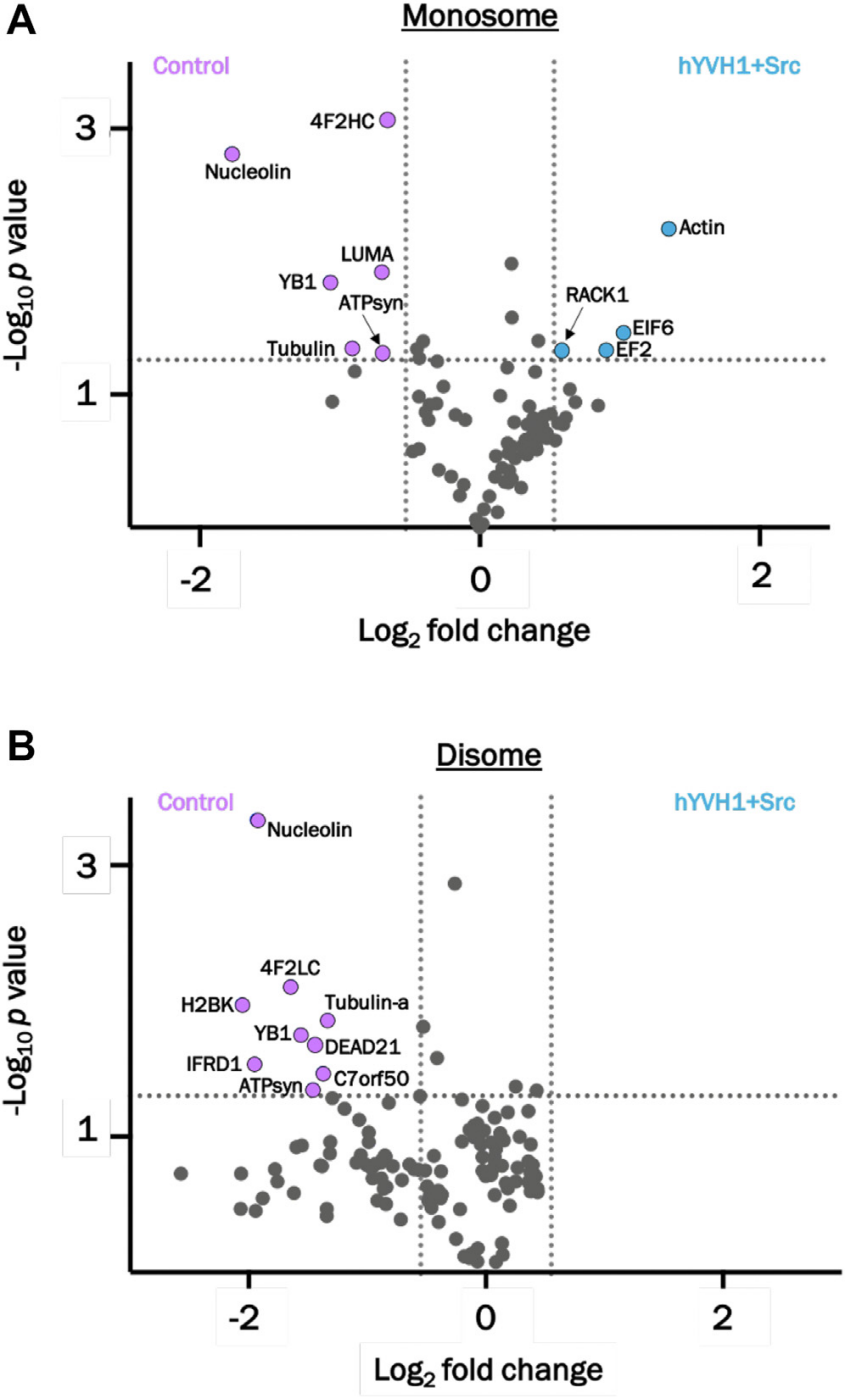
**Volcano plots indicating significant protein alterations for monosome and disome.** Comparison between control sample (*purple*) and hYVH1 + Src sample (*blue*). Significant proteins are indicated by the *colored circles*, whereas proteins deemed insignificant are colored in *gray*. *A*, monosome peak samples. *B*, disome peak samples. Only proteins identified with ≥3 peptides in all three biological replicates are represented. hYVH1, human YVH1.

To visually depict the protein alteration data, a volcano plot was constructed by comparing the −log_10_ *p* value from the Student's *t* test to the log_2_ fold change for the monosome and disome samples (Fig. 8). As expected, most of the identified proteins populate the base of the volcano plot indicating that the majority of ribosomal proteins identified in the monosome–disome samples were not significantly affected by coexpression of hYVH1 and Src. However, there are several significant protein alterations that are consistent with the working model that hYVH1 and Src coexpression enhances translational fitness (Table 1). The most statistically significant protein alteration was a reduction in the levels of nucleolin in both the monosome (3.4-fold higher in control) and disome samples (3.8-fold higher in control). Nucleolin is a multifunctional protein that has been shown to regulate chromosome condensation, DNA replication, and ribosomal RNA biogenesis (18, 19, 20). Moreover, a distinct pool of nucleolin has also been shown to associate with 3' and 5′ regions of select mRNA molecules at the fully mature ribosome, leading to enhanced translation or more commonly observed, repressed translation depending on the cellular context (19, 20). Another intriguing protein alteration was the reduction in nuclease-sensitive element-binding protein 1, better known as Y-box binding protein 1 (YB-1). YB-1 was found to be 2.1-fold higher in the control monosome sample and 2.9-fold higher in the control disome sample. YB-1 has been well characterized as a translational repressor in a wide variety of organisms (21, 22, 23, 24). Finally, reduced levels of the protein interferon-related developmental regulator 1 (IFRD1) in the disome sample was reproducibly detected in response to hYVH1–Src coexpression (3.8-fold higher in control). The role of IFRD1 in translational regulation is currently unclear; however, a close homolog, IFRD2, has been shown to induce translationally inactive ribosomes by associating with ribosomes that have a tRNA bound to a noncanonical site, suggesting that IFRD family members participate in repressing translation at the elongation stage (25, 26). These proteomic findings suggest that overexpression of hYVH1 and Src may attenuate the formation of disome ribosomal species that represent stalled intermediates through the regulation of associating factors that promote translational repression or ribosomal pausing.

**Table 1:** Significant proteome alterations of translational proteins

Protein	Role in translation	FC—monosome	FC—disome
Higher in control			
Nucleolin	Associates with 3′ and 5′ ends of mRNA to regulate translation	3.4	3.8
YB-1	mRNA-binding protein that promotes translational repression	2.1	2.9
IFRD1	Homolog of IFRD2 induces translationally inactive ribosomes	NS	3.8
Higher in hYVH1 + Src			
RACK1	40S Scaffold protein, translational fidelity, Src substrate	1.5	NS
EIF6	Regulates translational initiation, antiassociation factor	2.0	NS
EF2	Aminoacyl-tRNA and mRNA translocation, translational elongation	1.7	NS
F-actin	Required for local translation, associates with EF2	2.4	NS

Abbreviation: FC, fold change; NS, not significant.

In addition to the reduction of select translational repressive factors at the ribosome, coexpression of hYVH1 and Src increased levels of a couple key proteins at the monosome that have been shown to enhance translational fitness (Table 1). One critical factor that was found at elevated amounts was the GTPase, elongation factor 2 (EF2) (Fig. 8*A*). EF2 mediates the transfer of aminoacyl-tRNAs and mRNAs through the ribosome during translational elongation (27). Moreover, levels of EF2 at the 80S monosome are indicative of active elongation because of the fact that ribosome-bound EF2 is inaccessible to its negative regulator EF2 kinase (28). Therefore, higher monosome levels of EF2 found in the hYVH1–Src coexpression sample are consistent with the model that hYVH1–Src coexpression induces ribosomal structures capable of increased translational fitness. Also, we observed a clear increase in actin levels in the monosome fraction (2.4-fold) in response to hYVH1 and Src expression (Table 1 and Fig. 8*A*). There have been reports that the actin cytoskeleton is a critical regulator of protein translation, physically associates with polysomes, and actively participates in supporting localized translation (29). It is also interesting to note that EF2 has been shown to interact with the actin cytoskeleton *in vitro* (30). Along the same lines, a notable twofold increase in eukaryotic translation initiation factor 6 (EIF6) in the hYVH1–Src coexpression sample is further evidence of an increased translational fitness response. EIF6 is a potent regulator of translational initiation through its ability to function as an antiassociation factor (31). EIF6 docks onto the 60S subunit at the 60S–40S binding interface and is postulated to prevent translationally inactive 80S intermediates (*e.g.*, 80S devoid of mRNA and stalled disomes) (32). This apparent checkpoint role can be alleviated through phosphorylation by PKC to activate 80S formation in response to sufficient growth conditions (31). This phosphorylation event is facilitated by the scaffold protein receptor of activated protein C kinase 1 (RACK1) that associates with the 40S subunit (31, 33). Interestingly, we observed RACK1 to also be upregulated in the monosome in response to hYVH1–Src coexpression (Fig. 8*A*), suggesting hYVH1–Src may positively affect the EIF6–RACK1 signaling pathway that is crucial for proper translational fidelity. Consistent with this finding is a previous study reporting that Src associates with and phosphorylates RACK1 (34), indicating there is precedence for Src being in close proximity to the RACK1 scaffold.

It is unclear if the proteome alterations observed with hYVH1–Src coexpression is the result of a moonlighting function of hYVH1 unrelated to its 60S ribosome biogenesis role or if the alterations are the result of increased recycling of hYVH1 because of Src-mediated tyrosine phosphorylation of hYVH1. The immunofluorescent microscopy data showing increased nuclear localization in response to Src phosphorylation of hYVH1 support the hypothesis of a recycling effect. However, it is also possible that once hYVH1 is released from the 60S ribosome following 60S ribosomal stalk association, it performs additional modulating activities that affect the monosome proteome and that Src-mediated tyrosine phosphorylation of Tyr^179^ facilitates shuttling hYVH1 back to the nucleus for another round of 60S ribosome maturation. While a substantial amount of work will be required to elucidate the mechanistic details of hYVH1–Src regulation of translational fitness, the quantitative MS efforts have discovered valuable proteome alterations that will serve as useful biomarkers for future studies. Moreover, it is important to consider these results in the context of already established observations that hYVH1 expression affects cellular outcomes, such as cell survival, cell cycle regulation, and cell growth (4, 5). Such pleiotropic effects coupled with our current findings lead us to postulate a working model that the role of hYVH1 in the maintenance of 60S ribosome biogenesis and translational fitness collectively impacts the synthesis of proteins critical for achieving cellular homeostasis.

## Experimental procedures

### Plasmid constructs

The plasmids encoding FLAG-hYVH1 variants have been described previously (3). Site-directed mutagenesis was performed to generate hYVH1 Y179F and Y179E. The forward and reverse primers used were the following: 5′-CCTCTAGTGCAATTTTTAAGCAATATCGTTTAC-3′ and 5′-GTAAACGATATTGCTTAAAAATTGCACTAGAGG-3′ for Y179F and 5′-CCTCTAGTGCAATTGAAAAGCAATATCGTTTAC-3′ and 5′-GTAAACGATATTGCTTTTCAATTGCACTAGAGG-3′ for Y179E and confirmed by automated DNA sequencing (ACGT Corp). The purification of recombinant hYVH1 C115S has been previously described (4, 6). Human myc-Src Y530F mammalian construct was generously provided by Dr Michel Tremblay (McGill University).

### Cell culture

HeLa cells (American Type Culture Collection) were grown and maintained as a monolayer in Dulbecco’s modified Eagle's medium Nutrient Mixture F12-HAM (Sigma–Aldrich; catalog no.: D8437), supplemented with 10% (v/v) fetal bovine serum (Sigma–Aldrich; catalog no.: F7942) and 1% (v/v) penicillin–streptomycin (Thermo Fisher; catalog no.: 15140122) at 37 °C and 5% CO_2_. For endogenous hYVH1 experiments, cells were serum starved for 24 h prior to stimulation with pervanadate (1 mM) and epidermal growth factor (10 ng/ml) for 20 min in the presence or the absence of the Src-1 inhibitor (20 μM) (MCE; catalog no.: HY101053). For overexpression experiments, cells were split 24 h prior to transfection into antibiotic-free media. Respective complementary DNAs were introduced *via* lipid-mediated transfection using linear polyethylenimine (Polysciences; catalog no.: 23966). Five hours post-transfection, cells were washed with PBS (Thermo Fisher; catalog no.: SH3002802) and incubated overnight in fresh media with antibiotics. For SUnSET puromycin incorporation experiments, cells were treated with puromycin (Sigma) (10 μg/ml) for 20 min prior to cell lysis. About 24 h post-transfection, cells were washed with cold PBS and lysed in buffer containing 50 mM Tris–HCl (pH 7.4), 1% Triton X-100, 150 mM NaCl, and 0.1% SDS, supplemented with protease inhibitors—1 mM PMSF (Sigma–Aldrich; catalog no.: 78830) and 10 μg/ml aprotinin (Sigma–Aldrich; catalog no.: A3428). Soluble lysates were separated from cell debris by centrifugation at 24,000*g* for 10 min at 4 °C.

For ribosomal profiling experiments, 24 h post-transfection, cells were treated with 100 μg/μl cycloheximide (CHX; Sigma–Aldrich; catalog no.: C1988) for 10 min at 37 °C. Subsequently, cells were washed two times with cold PBS supplemented with 100 μg/μl CHX. Before cell lysis, transfected cells were pooled from three 15 cm cell culture plates by the addition of 3 ml of PBS with 100 μg/μl CHX per plate, followed by scraping cells and aliquoting the cell suspension into one 15 ml conical tube. Cells were harvested by centrifugation at 500*g* for 10 min at 4 °C. Cells were lysed in buffer containing 20 mM Tris–HCl (pH 7.4), 100 mM KCl, 5 mM MgCl_2_, and 0.3% NP-40 in diethyl pyrocarbonate (DEPC; Sigma–Aldrich; catalog no.: D5758) H_2_O, supplemented with 1 mM PMSF, 10 μg/ml aprotinin, 100 μg/μl CHX, 2 mM DTT (Sigma–Aldrich; catalog no.: D9163), and 1% RNaseOUT (Invitrogen; catalog no.: 10777-019). Buffer solutions were made in Milli-Q H_2_O treated with DEPC. Soluble lysates were separated from cell debris by centrifugation at 24,000*g* for 10 min at 4 °C. Fresh lysates were then loaded onto sucrose gradients for ribosomal profiling (see later).

### Immunoprecipitation

FLAG-hYVH1 was isolated from cellular lysates using Anti-FLAG M2 Affinity Gel resin (Sigma–Aldrich; catalog no.: A2220) *via* a 3 h incubation on a nutator at 4 °C. Samples were washed three times in buffer containing 50 mM Tris–HCl (pH 7.4), 0.1% Triton X-100, 150 mM NaCl, and 0.1% SDS and subsequently resuspended in 6× SDS-PAGE loading dye for analysis by immunoblotting or Coomassie staining using Imperial Protein Stain. Endogenous hYVH1 was isolated from HeLa cells using a rabbit anti-hYVH1 antibody conjugated to protein A agarose kindly provided from the laboratory of Jack E. Dixon (University of California San Diego School of Medicine) (3). For the *in vitro* kinase assays, recombinant hYVH1, purified from *Escherichia coli* cells as described previously (4, 6), and WT recombinant Src (SignalChem) were incubated *in vitro*, or myc-Src Y530F was isolated from cellular lysates using an anti-Src antibody (Sigma–Aldrich; catalog no.: 05184) conjugated to protein A agarose, and the kinase assays were performed as previously described (35).

### Immunoblotting

Following SDS-PAGE analysis, proteins were transferred onto polyvinylidene difluoride membranes (Millipore; catalog no.: IPVH00010) at 100 V for 1 h using the Mini-PROTEAN system by Bio-Rad. Polyvinylidene difluoride membranes were blocked for 1 h at room temperature in either 5% skim milk or 5% bovine serum albumin (BSA; Sigma–Aldrich; catalog no.: A9647) on a platform rocker. Membranes were then incubated on a rocker overnight at 4 °C in respective primary antibodies, including mouse anti-FLAG M2 (Sigma–Aldrich; catalog no.: F3165), mouse antipuromycin (Sigma–Aldrich; catalog no.: MABE343), mouse anti-4G10 (Millipore; catalog no.: 05-1050), mouse anti-c-Myc (Santa Cruz; catalog no.: sc-40), mouse anti-P_0_ (Santa Cruz; catalog no.: sc-293260), and rabbit anti-S3 (Cell Signaling; catalog no.: 2579S). The following day, membranes were washed three times in 1× Tris-buffered saline and 0.1% Tween and then incubated on a rocker for 45 min at room temperature in the respective secondary antibodies, including goat anti-mouse-horseradish peroxidase (Sigma–Aldrich; catalog no.: A4416) and goat anti-rabbit-horseradish peroxidase (Bio-Rad; catalog no.: 170-6515). After three more washes in 1× Tris-buffered saline and 0.1% Tween, chemiluminescent images were obtained using SuperSignal West Femto Maximum Sensitivity Substrate (Thermo Fisher; catalog no.: 34095) on a Bio-Rad ChemiDoc Imaging System (catalog no.: 17001401).

### Immunofluorescence microscopy

HeLa cells were seeded at 50,000 cells/ml on eight-chamber slides with 500 μl chambers (BioBasic; catalog no.: SP41219) using Opti-MEM reduced serum media (Thermo Fisher; catalog no.: 31985-062). Transfection was achieved using Lipofectamine 3000 (Invitrogen; catalog no.: L3000) with 0.3 μg of FLAG-hYVH1 WT, Y179F, Y179E, FLAG-pCMV empty vector, and myc-Src Y530F in antibiotic-free media. Five hours post-transfection, cells were washed with PBS and incubated overnight in fresh media with antibiotics. For stress granule analysis, sodium arsenite (NaAsO_2_) treatment was carried out 23 h post-transfection by incubating cells with a final concentration of 0.5 mM NaAsO_2_ at 37 °C for 1 h. At 24 h post-transfection, cells were washed once with PBS and then fixed with 3.7% paraformaldehyde (Thermo Fisher; catalog no.: AAJ19943K2) for 15 min at room temperature. Cells were washed with PBS and then permeabilized using 0.15% Triton X-100 in PBS for 2 min at room temperature, followed by a final PBS wash. Cells were blocked with 5% BSA in PBS for 1 h at room temperature with gentle shaking. Primary antibody incubations were conducted for 1 h at room temperature in 1% BSA, using either mouse anti-FLAG M2 (Sigma–Aldrich; catalog no.: F3165) or goat anti-TIA-1 (Santa Cruz; catalog no.: sc-1751) gently shaking at room temperature. The cells were then washed three times with PBS and subsequently incubated in secondary antibody for 1 h at room temperature in 1% BSA, using either horse antimouse-fluorescein (Vector Labs; catalog no.: FI-2000) or rabbit antigoat-Texas Red (Vector Labs; catalog no.: TI-5000), respective to the primary antibody used. Cells were washed another three times with PBS and incubated for 2 min at room temperature with 0.5 mg/ml Hoechst 33342 stain (Invitrogen; catalog no.: H3570) diluted in PBS. After a final PBS wash, the slides were then allowed to dry for 15 min, followed by coverslip mounting with 50% glycerol and sealed on all edges with clear nail polish (Super Dry). Fluorescence microscopy images were obtained with a Leica DMI6000 using a 40× oil objective. Images were quantitatively analyzed using ImageJ, with the JACoP plugin used for determination of stress granule size and PCCs, and the subcellular localization percentages were determined by the Intensity Ratio Nuclei Cytoplasm plugin. Measurements were obtained on 30 cells per sample, totaled from three independent experiments. Statistical analysis was performed using the Student’s *t* test (Prism), with differences considered statistically significant at *p* values <0.05 (specific values shown with each relevant figure).

### Ribosomal profiling

Sucrose solutions were prepared using DEPC-treated Milli-Q H_2_O at 5% and 40% sucrose (Sigma–Aldrich; catalog no.: S7903) concentrations in a buffer of 20 mM Hepes (pH 7.4), 150 mM KCl, 15 mM MgCl_2_, 100 μg/μl CHX, and 1 mM DTT. Sucrose gradients were prepared by layering 5% sucrose solution into 17 ml Open-Top Polyclear centrifuge tubes (Seton Scientific; catalog no.: 7042), followed by addition of 40% sucrose solution to the bottom of the tube. Gradients were formed using BioComp Gradient Maker (model 108) with tube holder SW28.1, on settings short, 5 to 40% sucrose (w/v) with long caps. Once formed, gradients were stored at 4 °C for 1 h.

Equal amounts of lysate (equivalent absorbance at 260 nm of 10) were carefully layered on top of the sucrose gradients and ultracentrifuged at 167,000*g* at 4 °C for 3 h (Thermo Scientific; Sorvall SureSpin 630). Upon completion, gradients were loaded onto a piston gradient fractionator (BioComp Instruments; catalog no.: 157) and fractionated with the parameters of speed: 0.2 mm/s, distance: 85.0 mm, and number of fractions: 30. Throughout fractionation, absorbance was continually monitored at a wavelength of 254 nm using a UV Monitor (Bio-Rad; catalog no.: 731-8160), and a fraction collector was used to collect fractionated samples (Gilson; catalog no.: FC203B). Quantitation of the area under the curve was performed in ImageJ. Peak areas of the high polysome fractions were normalized to the corresponding 40S peak area. Measurements were obtained on all the independent experiments (n = 3) for the different samples, and statistical analysis was performed using the Student’s *t* test (Prism), with differences considered statistically significant at *p* values <0.05. Fractions were then stored at −20 °C until further analysis by immunoblotting or MS.

### In-gel trypsin digestion

Ribosomal fractions were resuspended in 2× SDS-PAGE loading dye and loaded onto a 15% SDS-PAGE gel. Samples were run using a “short stack” method, where the gel running is stopped once all the sample has entered the resolving layer of the gel (30 min). Gels were stained overnight with Imperial Protein Stain (Thermo Fisher; catalog no.: 24615) and destained the following day with Milli-Q H_2_O. The entire sample area was excised from the gel with a scalpel blade and cut into small pieces. Gel pieces were then destained further in a 2:1 ACN (Honeywell Burdick & Jackson; catalog no.: 015-4):50 mM ammonium bicarbonate (AB; Thermo Fisher; catalog no.: A643-500) solution, at 37 °C for 15 min, and repeated a second time. Gel pieces were subsequently dehydrated by addition of 100% ACN, vortexed, and dehydrated a second time. The shrunken semidried gel pieces were then dried completely *via* vacuum centrifugation (Thermo Fisher) for 15 min. Gel pieces were rehydrated in trypsin digestion buffer, consisting of 15 ng/μl sequencing-grade modified trypsin (Promega; catalog no.: V5111), which specifically cleaves proteins C terminal to Arg and Lys residues, diluted in 50 mM AB. Samples were incubated on ice for 30 min, before the addition of more AB to ensure gel pieces were fully immersed and placed in a shaking incubator overnight at 37 °C. Peptides were extracted from the gel pieces by adding 150 μl of extraction buffer, consisting of 2:1 ACN:5% formic acid (FA; Thermo Fisher; catalog no.: A117-50), incubated at 37 °C for 15 min, and this step then repeated a second time. Peptides were concentrated *via* vacuum centrifugation for 1.5 h and reconstituted in 0.15% TFA (Thermo Fisher; catalog no.: 28904). Peptides were purified using Oasis HLB 1 cc Extraction Cartridge columns (Waters; catalog no.: 186000383). Initially, columns were activated with 100% ACN, followed by equilibration with 0.15% TFA, repeated five times total. On the fifth equilibration, 1% ACN was added to the 0.15% TFA solution. Peptide solutions were then loaded onto their respective columns, followed by three washes with 0.15% TFA. Elution of peptides was performed in a stepwise manner, with the first elution being 20:80 ACN:0.15% TFA, second elution 50:50 ACN:0.15% TFA, and the third and fourth elutions being 80:20 ACN:0.15% TFA. These purified peptides were then concentrated again *via* vacuum centrifugation for 2 h, reconstituted in 0.1% FA in MS grade water (Honeywell Burdick & Jackson; catalog no.: 365-4), and kept at −20 °C until MS analysis.

### MS

Peptides were loaded onto a 1.8 μm HSS T3 75 μm × 150 mm reverse-phase column (Waters) at a flow rate of 0.3 μl/min *via* the nanoAcquity UPLC autosampler. Peptide separation was achieved using a gradient consisting of mobile phase A (0.1% FA in water) and mobile phase B (ACN with 0.1% FA). Equilibration and loading conditions used was a 97:3 solvent ratio (mobile A:B). Peptide elution was achieved using a 90 min gradient (3–30% B for 55 min, 30–50% B for 25 min, and 85% B for 10 min) and directly sprayed into a SYNAPT G2-S*i* mass spectrometer (Waters) operating with a 3 kV capillary voltage and a 30 V cone voltage. The high definition MS^E^ operating mode was utilized consisting of DIA with ion mobility separation activated using a wave speed of 650 m/s. High-definition MS^E^ data were measured using low energy scans at 4 eV and high energy scans at 20 to 45 eV in positive high-resolution mode, scanning from 50 to 2000 *m/z* at a rate of 0.8 s. Calibration was using [Glu1]-fibrinopeptide B (50 fmol/μl) in the lock mass channel at *m/z* 785.8427 for a doubly charged positive ion. Raw data were collected using Mass Lynx (Waters, version 4.1).

For ribosome quantitative proteomics, raw data from three biological replicates for each condition were processed using the Progenesis QI (Nonlinear Dynamics) software package for chromatogram alignments and normalization adjustments. Label-free quantitation was accomplished by the Hi-3 method (36), which uses the intensity of the three most abundant peptides per protein to calculate relative abundance. Protein identification was accomplished utilizing the human UniProtKB/SwissProt database (26,465 proteins), acquired July 10, 2018. The following parameters were used to process the raw data: a low-energy noise reduction of 135 counts, a high-energy noise reduction threshold of 30 counts, and an intensity threshold of 750 counts. Lock mass calibration correction occurred postacquisition using Glu-fib as a standard. The following parameters were used for protein identification: a minimum of three fragment ions per peptide, a minimum of seven fragment ions per protein, and a minimum of one unique peptide match per protein. The mass tolerance for precursor and fragment ions was set to “Auto,” and peptide scores less than 5.0 were discarded. The maximum false discovery rate used was 1% using a decoy reverse database. No fixed modification was considered. A maximum of one missed cleavage following trypsin digestion was permitted, and the variable modification of methionine oxidation +15.9949 was included.

## Data availability

The MS proteomics data have been deposited to the ProteomeXchange Consortium *via* the PRIDE [1] partner repository with the dataset identifier PXD030982.

Username: reviewer_pxd030982@ebi.ac.uk

Password: KaIXkv6t.

## Supporting information

This article contains supporting information.

## Conflict of interest

The authors declare that they have no conflicts of interest with the content of this article.
