# Supplementary material for: Src-mediated phosphorylation of the ribosome biogenesis factor hYVH1 affects its localization, promoting partitioning to the 60S ribosomal subunit
Source: J Biol Chem. 2022 Nov 10;298(12):102679. doi: 10.1016/j.jbc.2022.102679 (PMC9731860; doi:10.1016/j.jbc.2022.102679)
Supplement: Supplemental Fig S1 [file mmc4.pdf]

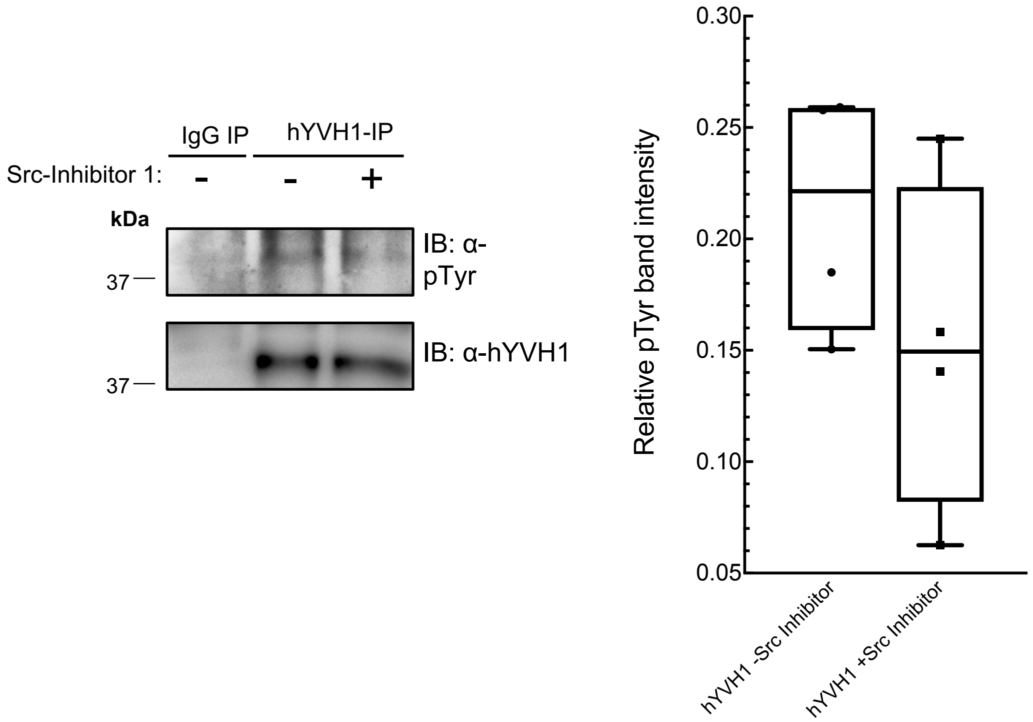

Figure S1 – Endogenous tyrosine phosphorylation of hYVH1. *A,* HeLa cells serum starved for 24 hrs were treated with 1mM pervanadate and EGF stimulation (10 ng/ml) for 20 min. Cellular lysates were subjected to anti-hYVH1 immunoprecipitation and analyzed by anti-phosphotyrosine (α-pTyr) and anti-hYVH1 immunoblotting. As a negative control, HeLa cells were immunoprecipitated with resin containing rabbit IgG (left lane). HeLa cells were also treated with either DMSO vehicle control (-) or 20 μM of Src-1 inhibitor for 1hr (+). Shown are representative blots from four independent replicates. *B,* Densitometry analysis of endogenous hYVH1 phosphotyrosine levels in the absence or presence of the Src inhibitor. Each biological replicate was normalized to levels of total hYVH1 and densitometry analysis was performed using the Image J software package. Values are mean ± standard deviation of the mean of four independent experiments.
