# Supplementary material for: Src-mediated phosphorylation of the ribosome biogenesis factor hYVH1 affects its localization, promoting partitioning to the 60S ribosomal subunit
Source: J Biol Chem. 2022 Nov 10;298(12):102679. doi: 10.1016/j.jbc.2022.102679 (PMC9731860; doi:10.1016/j.jbc.2022.102679)
Supplement: Supplemental Fig S2 [file mmc5.pdf]

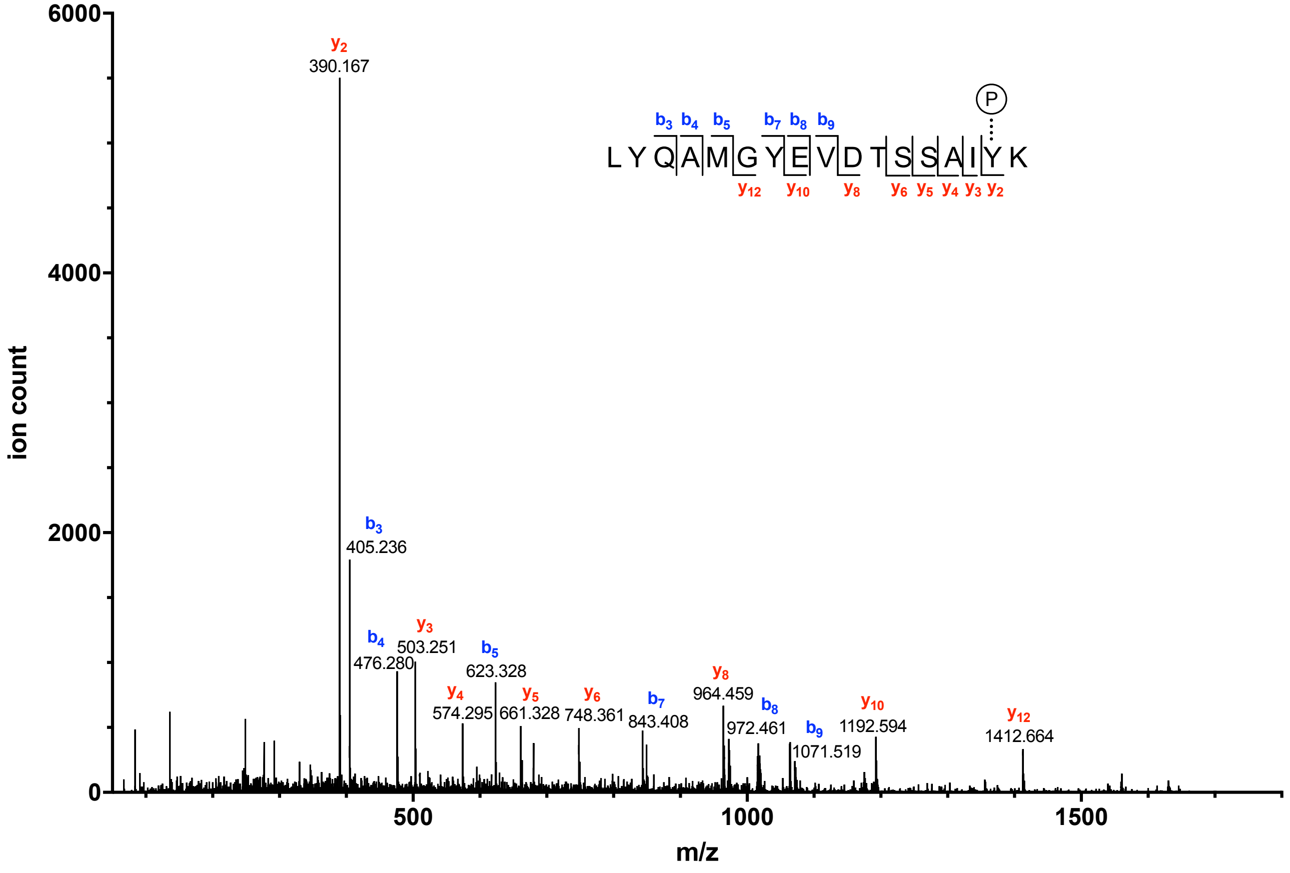

Figure S2 – Identification of Tyr 179 as a novel hYVH1 phosphorylation site. HeLa cells expressing FLAG-hYVH1 and myc-Src Y530F were subjected to FLAG immunoprecipitation and trypsin digestion. Mass spectrometry analysis identified a monophosphorylated tryptic peptide at m/z 1017.9^{+2} corresponding to amino acids 164-180. MS/MS analysis unambiguously mapped the phosphorylation site to Tyr179.
