# Supplementary material for: Src-mediated phosphorylation of the ribosome biogenesis factor hYVH1 affects its localization, promoting partitioning to the 60S ribosomal subunit
Source: J Biol Chem. 2022 Nov 10;298(12):102679. doi: 10.1016/j.jbc.2022.102679 (PMC9731860; doi:10.1016/j.jbc.2022.102679)
Supplement: Supplemental Fig S3 [file mmc6.pdf]

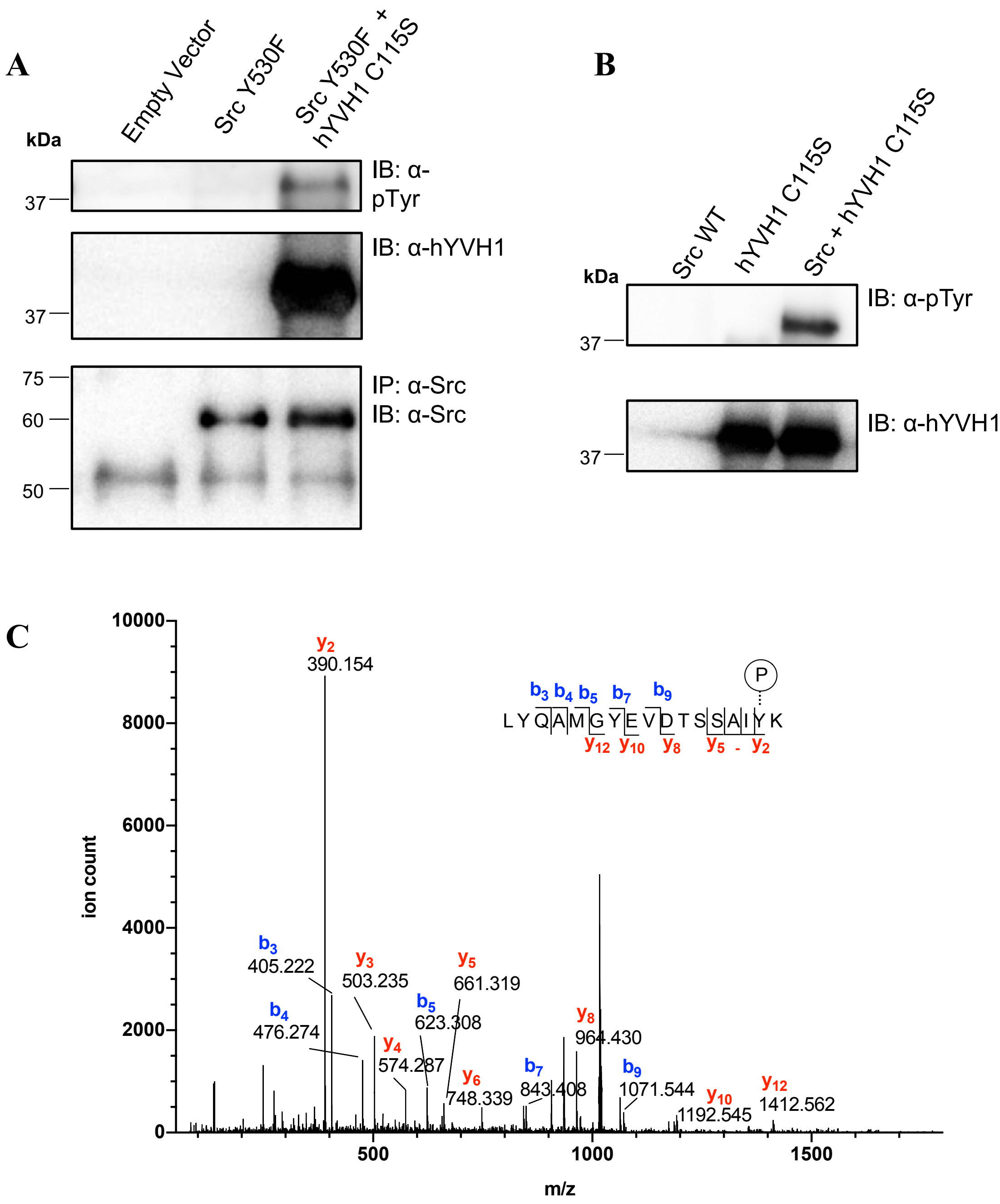

Figure S3 – Src phosphorylates recombinant hYVH1 *in vitro*. *A,* HeLa cells expressing myc-Src Y530F were subjected to anti-Src immunoprecipitation and an *in vitro* kinase assay was performed using recombinant hYVH1 C115S purified from *E.coli* cells. Following the *in vitro* kinase assay, samples were analyzed by anti-phosphotyrosine immunoblotting (α-pTyr). *B,* In vitro kinase assay using recombinant wild type Src and recombinant hYVH1 C115S. Following the kinase assay phosphorylation of hYVH1 was analyzed by anti-phosphotyrosine immunoblotting (α-pTyr). *C,* Representative MS/MS fragment ion spectrum of a monophosphorylated tryptic peptide at m/z 1017.9^{+2} corresponding to amino acids 164-180 of hYVH1. Analysis of the fragmentation pattern unambiguously mapped the phosphorylation site to Tyr179.
